# Previously Unrecognized Congenital Hypogonadotropic Hypogonadism in an 89-Year-Old Man: A Case Report

**DOI:** 10.7759/cureus.116742

**Published:** 2026-09-22

**Authors:** Mafalda Ferreira, Patrícia O Saraiva, Franciso Nunes, João Cardoso, Maria Eugénia André

**Affiliations:** 1 Internal Medicine, Unidade Local de Saúde de Castelo Branco, Castelo Branco, PRT

**Keywords:** bilateral congenital cryptorchidism, bilateral gynecomastia, central hypogonadism, congenital hypogonadotropic hypogonadism, micropenis

## Abstract

Congenital hypogonadotropic hypogonadism (CHH) is a rare disorder typically recognized during adolescence because of absent or incomplete pubertal development. We report the case of an 89-year-old man admitted for an ischemic stroke whose physical examination revealed a high-pitched voice, markedly sparse facial and axillary hair, bilateral gynecomastia, a markedly reduced penile size clinically suggestive of micropenis, and nonpalpable testes in the scrotum.

Hormonal evaluation showed a total testosterone concentration below 7.00 ng/dL, luteinizing hormone below 0.07 IU/L, and follicle-stimulating hormone of 0.83 IU/L, consistent with severe central hypogonadism. Ultrasonography demonstrated both testes within the inguinal canals with reduced testicular size. Magnetic resonance imaging (MRI) of the sellar region showed no structural hypothalamic-pituitary abnormality. Although advanced age and acute systemic illness may have contributed to further functional suppression of the hypothalamic-pituitary-gonadal axis, these factors could not account for the developmental genital abnormalities. Integration of the phenotypic, biochemical, and imaging findings supported a clinical diagnosis of previously unrecognized CHH.

This case illustrates an exceptionally late recognition of CHH and highlights the diagnostic value of developmental phenotypic features in identifying a congenital disorder that may remain unrecognized for decades.

## Introduction

Congenital hypogonadotropic hypogonadism (CHH) is a rare disorder caused by impaired secretion or action of gonadotropin-releasing hormone (GnRH), resulting in deficient luteinizing hormone (LH) and follicle-stimulating hormone (FSH) secretion and consequent sex steroid deficiency [1,2]. CHH is clinically and genetically heterogeneous. When associated with anosmia or hyposmia, it is classified as Kallmann syndrome, whereas cases with preserved olfaction are referred to as normosmic CHH. Its genetic architecture is complex, with both monogenic and oligogenic forms described, although a substantial proportion of patients remain without an identified molecular etiology [1-4].

In males, CHH typically presents with absent or incomplete pubertal development, reduced testicular volume, underdevelopment of secondary sexual characteristics, sexual dysfunction, and infertility [1-3]. In more severe phenotypes, however, gonadotropin deficiency is already present during early developmental stages. Activity of the hypothalamic-pituitary-gonadal axis during late fetal life and its transient postnatal activation, known as mini-puberty, play important roles in penile growth, testicular descent, and Leydig and Sertoli cell maturation [5,6]. Accordingly, micropenis and/or cryptorchidism are important clinical markers of early gonadotropin deficiency and provide evidence supporting a congenital etiology [5,6]. Comparative studies have further shown that cryptorchidism and micropenis are more frequent in men with CHH than in those with acquired hypogonadotropic hypogonadism [7].

The diagnosis of CHH is based on the combination of clinical features and biochemical evidence of hypogonadotropic hypogonadism, while the differential diagnosis includes acquired functional or structural causes of central hypogonadism and, in selected patients, other congenital disorders of sexual development. Genetic testing may provide additional support and help identify an underlying molecular cause, although a negative genetic result does not exclude CHH because of its marked genetic heterogeneity [1-4].

CHH is usually diagnosed during adolescence because of absent or inadequate pubertal progression, although it may be recognized later during the evaluation of sexual dysfunction or infertility [1-3]. Although CHH is rare, delayed or missed diagnosis may occur, particularly when pubertal or reproductive history is unavailable or when the clinical presentation is not recognized during adolescence. Initial recognition at a very advanced age is therefore exceptional and poses particular diagnostic challenges. Aging, acute or chronic systemic illness, frailty, malnutrition, and certain medications may contribute to functional suppression of the hypothalamic-pituitary-gonadal axis, leading to reduced testosterone concentrations with low or inappropriately normal gonadotropin levels [8,9]. In this setting, biochemical findings alone may be insufficient to distinguish acquired functional hypogonadism from congenital gonadotropin deficiency. Diagnostic interpretation therefore relies on integrating the hormonal profile with the clinical history and, importantly, with phenotypic features established during development [7,9].

## Case presentation

We report the case of an 89-year-old man with a history of arterial hypertension and dyslipidemia, treated with perindopril/amlodipine 10 mg/10 mg and simvastatin 20 mg, who was admitted with an ischemic stroke after approximately five days of language disturbance. During hospitalization, the language impairment persisted and was accompanied by episodes of delirium, limiting the ability to obtain a reliable medical history.

On admission, physical examination revealed several features suggestive of longstanding androgen deficiency, including a high-pitched voice, markedly sparse facial and axillary hair, bilateral gynecomastia (Figure 1), reduced penile dimensions (estimated flaccid penile length of 1 cm) (Figure 2), and nonpalpable testes within the scrotum. Penile length was not formally measured using a standardized technique, and erect penile length was not assessed. A simple bedside assessment of olfaction using familiar odors did not reveal an apparent olfactory deficit; however, formal psychophysical olfactory testing was not performed. There was no documented history of a previous endocrine diagnosis or treatment with testosterone or gonadotropins.

**Figure 1:**
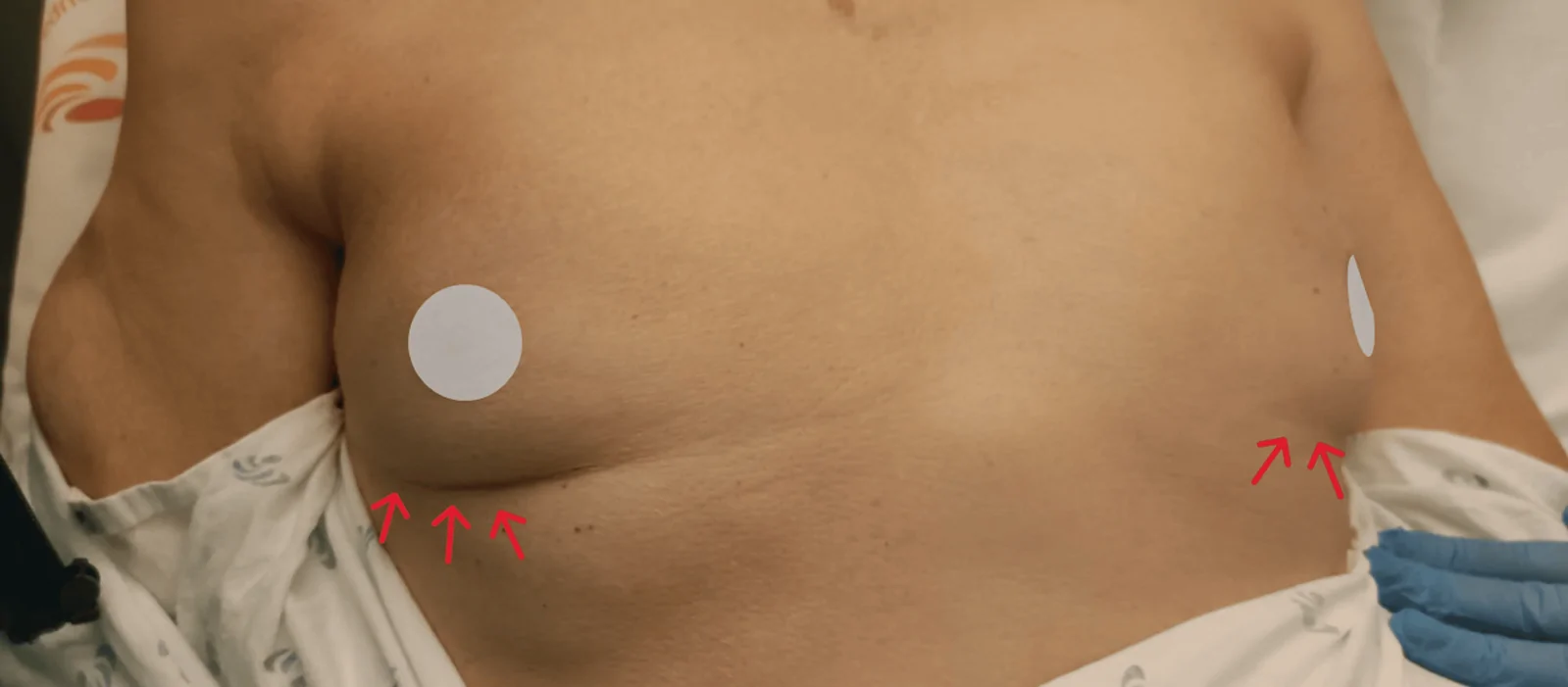
Phenotypic findings on physical examination: gynecomastia (red arrows)

**Figure 2:**
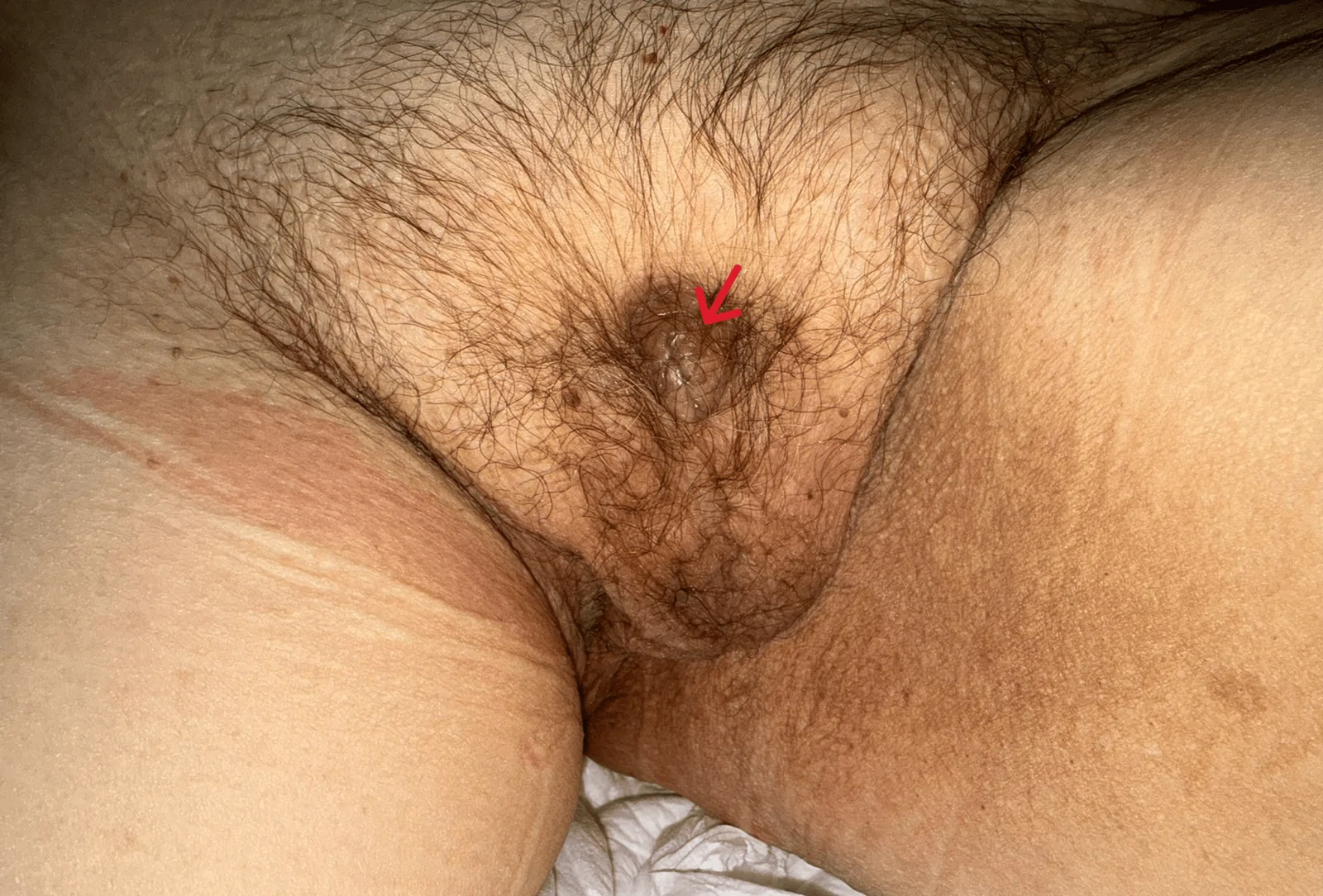
Phenotypic findings on physical examination: penis of reduced dimensions (red arrow)

The neurological abnormalities precluded reliable characterization of pubertal development and sexual and reproductive history, as well as a detailed family history of delayed puberty or infertility. It was also not possible to determine whether the cryptorchidism had previously been recognized or treated. According to information provided by family members, the patient had experienced difficulty achieving conception and had no biological children.

Hormonal evaluation performed during hospitalization showed a total testosterone level below 7.00 ng/dL, LH below 0.07 IU/L, and FSH of 0.83 IU/L. Cytogenetic analysis demonstrated a 46,XY karyotype, with no chromosomal abnormalities identified in the metaphases examined (Table 1).

**Table 1:** Hormonal evaluation and cytogenetic analysis LH: luteinizing hormone, FSH: follicle-stimulating hormone.

Hormone	Value	Reference value
Testosterone	<7.00 ng/dL	86.43-788.22 ng/dL
LH	<0.07 IU/L	3.1-34.6 IU/L
FSH	0.83 IU/L	1.4-18.1 IU/L
Karyotype	46, XY (no chromosomal abnormalities identified in the metaphases examined)	

Scrotal ultrasonography identified both testes within the inguinal canals. Both were reduced in size and showed a hypoechoic echotexture, without focal lesions or masses.

Magnetic resonance imaging (MRI) of the sellar region showed no relevant structural abnormalities of the pituitary gland or pituitary stalk and no mass lesion within the hypothalamic-pituitary region.

Taken together, the clinical, biochemical, and imaging findings supported a clinical diagnosis of previously unrecognized CHH.

Testosterone replacement therapy was not initiated during hospitalization because of the recent ischemic stroke.

## Discussion

Central hypogonadism is characterized by low testosterone concentrations in the presence of low or inappropriately normal gonadotropin levels [1-3,8]. In the present case, total testosterone was below 7.00 ng/dL, LH was almost undetectable, and FSH was reduced, establishing a biochemical pattern of severe central hypogonadism. MRI of the sellar region showed no structural abnormalities of the pituitary gland or pituitary stalk. However, normal imaging does not, by itself, distinguish a hypothalamic from a pituitary defect. Although hormonal testing was performed during acute illness and was not confirmed by a second morning testosterone measurement under clinically stable conditions, the biochemical findings acquire greater diagnostic significance when interpreted in conjunction with the phenotype [8]. The severity of the hormonal deficiency alone cannot establish a congenital etiology; however, its association with developmental genital abnormalities strongly supports a congenital origin over purely functional or acquired causes [1-3,8].

Bilateral cryptorchidism and micropenis were the most informative clinical findings supporting a congenital origin. Activity of the hypothalamic-pituitary-gonadal axis during fetal life is essential for genital androgenization and testicular descent. Following birth, transient activation of this axis during mini-puberty is characterized by physiological increases in LH, FSH, and testosterone and contributes to penile growth and testicular maturation [5,6,10]. In severe CHH, gonadotropin deficiency during these early developmental periods may result in micropenis and/or cryptorchidism, making these findings important clinical markers of early gonadotropin deficiency [5,6,10]. Comparative studies have further shown that cryptorchidism and markedly reduced testicular volume are more frequent in men with CHH than in those with acquired hypogonadotropic hypogonadism [7].

A major diagnostic challenge in this patient was the possible coexistence of functional suppression of the hypothalamic-pituitary-gonadal axis. Aging and acute systemic illness may both be associated with reduced testosterone concentrations and an inadequate gonadotropin response [8,9] and may therefore have contributed to the severity of the biochemical abnormalities observed. These factors, however, cannot account for bilateral cryptorchidism or markedly impaired penile development, both of which necessarily predated the acute illness. Accordingly, the distinction between CHH and functional hypogonadism was not based solely on hormone concentrations, but rather on their concordance with a phenotype indicating gonadotropin deficiency from early developmental stages [1,2,7].

The absence of a structural hypothalamic-pituitary lesion on MRI made an acquired structural cause of central hypogonadism less likely, although normal imaging cannot exclude nonstructural hypothalamic or pituitary dysfunction. Prolonged retention of the testes within the inguinal canals may have contributed to their reduced size and abnormal echotexture. Isolated primary testicular dysfunction, however, would generally be expected to result in compensatory elevation of gonadotropin levels, which was not observed [2,3]. Ultrasonography also confirmed the presence of bilateral testicular tissue, thereby excluding anorchia.

Cytogenetic analysis demonstrated a 46,XY karyotype, with no chromosomal abnormalities identified in the metaphases examined. This finding should be regarded as complementary to the diagnostic evaluation: it did not identify an alternative sex-chromosome abnormality that could account for the phenotype, but it does not itself support or establish the diagnosis of CHH. The genetic basis of CHH is highly heterogeneous, and a substantial proportion of patients remain without an identified molecular etiology even after targeted genetic investigation [1,4]. Nevertheless, the differential diagnosis of a 46,XY individual with cryptorchidism and penile underdevelopment is broader than CHH. Other congenital disorders of sexual development, including disorders affecting androgen production or action, cannot be completely excluded in the absence of additional endocrine, functional, or molecular investigations. Thus, the present diagnosis should be regarded as a clinically supported diagnosis of CHH rather than a molecularly confirmed etiology.

A simple clinical assessment of olfaction did not reveal an apparent deficit. This finding is compatible with a normosmic phenotype but should be interpreted cautiously because standardized psychophysical olfactory testing was not performed. Olfactory function in CHH spans a spectrum from normosmia to hyposmia and anosmia, and subjective or bedside assessment may not accurately reflect objectively measured olfactory function [2,11]. The available findings therefore do not allow definitive classification of the olfactory subtype.

Initial recognition of CHH at 89 years of age is exceptional, as the disorder is usually diagnosed during adolescence because of absent or incomplete pubertal development or later during the evaluation of sexual dysfunction or infertility [1-3]. In this case, the neurological abnormalities prevented reliable reconstruction of the patient's pubertal, sexual, and reproductive history, representing an important limitation. Information obtained from family members, including a history of difficulty achieving conception and the absence of biological children, was compatible with possible longstanding reproductive impairment but was insufficient to establish retrospective infertility. In this setting, developmental anatomical findings provided particularly valuable temporal information because bilateral cryptorchidism and reduced penile dimensions could not be attributed to a hormonal disorder acquired in advanced age [7,10].

Untreated CHH may be associated with sexual and reproductive dysfunction, reduced bone mass, and other systemic consequences of prolonged sex steroid deficiency [2,12,13]. Testosterone replacement therapy was not initiated during hospitalization. The Endocrine Society guideline recommends against initiating testosterone therapy in men who have experienced a stroke within the preceding six months [8]. Moreover, routine testosterone therapy is not recommended for all men aged 65 years or older solely on the basis of low testosterone concentrations; treatment decisions should instead be individualized according to symptoms, comorbidities, and the balance of potential benefits and risks [8].

Thus, in this patient, recognition of CHH was primarily of diagnostic and explanatory value, providing a coherent framework for a longstanding phenotype without warranting immediate androgen replacement.

## Conclusions

CHH may remain unrecognized until an exceptionally advanced age. Careful clinical history-taking and detailed physical examination are essential for its recognition, particularly when developmental phenotypic findings provide temporal information that cannot be reliably reconstructed from the medical history. In this case, micropenis and bilateral cryptorchidism strongly supported a congenital origin and could not be explained by an acquired hormonal disorder developing in advanced age.

The diagnosis of CHH was therefore primarily of diagnostic and explanatory value, providing a coherent interpretation of a longstanding phenotype without implying an immediate need for treatment. This case highlights the importance of developmental phenotypic features in distinguishing congenital from acquired forms of central hypogonadism and underscores the value of a comprehensive physical examination even when the diagnosis is incidental and unrelated to the reason for hospitalization.
